# Zinc coordination is essential for the function and activity of the type II secretion ATPase EpsE

**DOI:** 10.1002/mbo3.376

**Published:** 2016-05-10

**Authors:** Chelsea S. Rule, Marcella Patrick, Jodi L. Camberg, Natalie Maricic, Wim G. Hol, Maria Sandkvist

**Affiliations:** ^1^ Department of Microbiology and ImmunologyUniversity of Michigan Medical SchoolAnn ArborMichigan; ^2^ Division of Infectious DiseasesDepartment of MedicineUniversity of Maryland School of MedicineRockvilleMaryland; ^3^ Department of BiochemistryBiomolecular Structure CenterUniversity of WashingtonSeattleWashington; ^4^ Present address: AddgeneCambridgeMassachusetts; ^5^ Present address: Department of Cell and Molecular BiologyUniversity of Rhode IslandKingstonRhode Island

**Keywords:** ATPase, tetracysteine, type II secretion, zinc.

## Abstract

The type II secretion system Eps in *Vibrio cholerae* promotes the extracellular transport of cholera toxin and several hydrolytic enzymes and is a major virulence system in many Gram‐negative pathogens which is structurally related to the type IV pilus system. The cytoplasmic ATPase EpsE provides the energy for exoprotein secretion through ATP hydrolysis. EpsE contains a unique metal‐binding domain that coordinates zinc through a tetracysteine motif (CXXCX_29_CXXC), which is also present in type IV pilus assembly but not retraction ATPases. Deletion of the entire domain or substitution of any of the cysteine residues that coordinate zinc completely abrogates secretion in an EpsE‐deficient strain and has a dominant negative effect on secretion in the presence of wild‐type EpsE. Consistent with the in vivo data, chemical depletion of zinc from purified EpsE hexamers results in loss of in vitro ATPase activity. In contrast, exchanging the residues between the two dicysteines with those from the homologous ATPase XcpR from *Pseudomonas aeruginosa* does not have a significant impact on EpsE. These results indicate that, although the individual residues in the metal‐binding domain are generally interchangeable, zinc coordination is essential for the activity and function of EpsE.

## Introduction

The type II secretion (T2S) system mediates the extracellular transport of virulence factors, such as toxins and hydrolytic enzymes, in many Gram‐negative pathogens (Sandkvist 2001; Cianciotto 2005; Korotkov et al. 2012). *Vibrio cholerae* uses the T2S system to secrete cholera toxin, which is largely responsible for the severe diarrhea that characterizes cholera as well as at least 20 other proteins such as proteases, chitinases, lipases, and biofilm matrix proteins (Overbye et al. 1993; Connell et al. 1998; Davis et al. 2000; Sikora et al. 2011; Johnson et al. 2014). Type II‐secreted proteins first cross the inner membrane using the Sec or Tat general export pathways, signal sequences are then removed, and these proteins are subsequently transported across the outer membrane via T2S (Pugsley 1993; Voulhoux et al. 2001). Type II‐secreted factors are important for nutrient acquisition and/or modulating the surroundings to benefit the bacteria in both the aquatic environment as well as in the human small intestine (Sandkvist 2001; Cianciotto 2005; Sikora 2013).

The T2S apparatus in *V. cholerae* spans both the inner and outer membranes and is composed of 12 Eps (*e*xtracellular *p*rotein *s*ecretion) proteins (denoted EpsC through EpsM) and PilD (Overbye et al. 1993; Sandkvist et al. 1997; Marsh and Taylor 1998; Fullner and Mekalanos 1999). The cytoplasmic ATPase EpsE is associated with the inner membrane through interactions with the bitopic inner membrane protein EpsL (Sandkvist et al. 1995; Abendroth et al. 2005). EpsE acts as a molecular motor to provide the energy for exoprotein secretion through ATP hydrolysis (Camberg and Sandkvist 2005). A recent study from our laboratory indicates that EpsL may provide a molecular link between EpsE and the major pseudopilin component EpsG. Protein–protein interactions between EpsG and EpsL were established through chemical crosslinking and coimmunoprecipitation followed by immunoblotting for EpsG or EpsL. EpsG prepilin processing by PilD is required for this EpsL interaction, although no other T2S proteins are necessary. The results suggest that the energy produced during ATP hydrolysis by EpsE may be transduced through EpsL to the major pseudopilin EpsG to power pseudopilus assembly for T2S (Gray et al. 2011).

**Figure 1:**
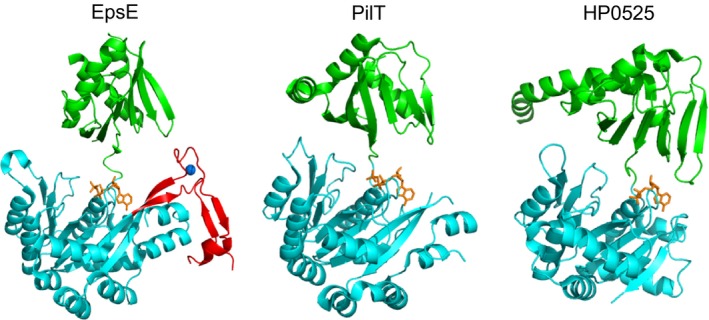
Structural comparison of type II/IV secretion ATPases. The structures of the *Vibrio cholerae* T2S ATPase EpsE (left, PDB 1P9W), the *Pseudomonas aeruginosa* type IV pilus retraction ATPase PilT (center, PDB 3JVV), and the *Helicobacter pylori* type IV secretion ATPase HP0525 (right, PDB 1G6O) are shown. N‐terminal domains are colored green, C‐terminal domains in cyan, and the C_M_ domain in EpsE is displayed in red with zinc represented as a blue sphere. Nucleotide is shown in orange.

EpsE belongs to a large family of type II/IV secretion ATPases, including those involved in protein secretion, type IV pilus (T4P) biogenesis, competence, and archaeal flagella (archaellum) assembly (Planet et al. 2001). Family members consist of two distinct domains: the N‐terminal domain (NTD) and the C‐terminal domain (CTD), which are connected by a short flexible linker (Robien et al. 2003; Satyshur et al. 2007; Misic et al. 2010; Rose et al. 2011; Reindl et al. 2013). The CTD contains the conserved ATP‐binding motifs that collectively form the nucleotide‐binding pocket, including Walker A and B motifs and Asp and His boxes (Robien et al. 2003; Chiang et al. 2008). Within this family, EpsE and other T2S ATPases as well as ATPases required for T4P assembly form the subfamily of GspE/PilB ATPases, alternatively referred to as pilus assembly ATPases (Planet et al. 2001). Members of this subfamily contain a unique domain called the C‐terminal metal‐binding domain (C_M_) that coordinates zinc through a tetracysteine motif (CXXCX_21–40_CXXC) (Fig. 1; Planet et al. 2001; Robien et al. 2003; Camberg and Sandkvist 2005). The C_M_ domain is notably absent among T4P retraction ATPases such as PilT and PilU (Whitchurch and Mattick 1994; Robien et al. 2003; Satyshur et al. 2007; Misic et al. 2010). The tetracysteine motif is conserved among all T2S ATPases except for *Xylella fastidiosa* XpsE and *Xanthomonas campestris* XpsE (Robien et al. 2003). The EpsE C_M_ domain spans residues C397–C433 in EpsE, with the amino acids in between the two dicysteines forming a hairpin turn or loop. The C_M_ loop residues share low sequence homology (~30%) between homologous T2S ATPases (Robien et al. 2003; Camberg and Sandkvist 2005). Structural analysis shows that the C_M_ domain is located on the outside of the EpsE hexamer (Lu et al. 2013).

Zinc‐coordinating domains have been implicated in diverse roles such as stability, protein–protein interactions, and regulation of activity (Fekkes et al. 1999; Jakob et al. 2000; Salzer et al. 2014). The importance of cysteine residues to the C_M_ domain of GspE/PilB ATPases has been previously suggested by other studies (Possot and Pugsley 1997; Salzer et al. 2014). The T2S ATPase PulE from *Klebsiella oxytoca* contains a tetracysteine motif similar to EpsE, and loses the ability to support secretion of pullulanase when at least two adjacent cysteines are replaced with serines. However, the insolubility of native PulE and lack of protein purification techniques prevented study of PulE in vitro (Possot and Pugsley 1997). *Thermus thermophilus* PilF is an ATPase involved in T4P biogenesis and DNA uptake, and was recently shown to require zinc for stability of PilF hexameric complexes, but not for ATPase activity in vitro. Additionally, cysteine residues were important for PilF's role in piliation at high temperatures, but not for the transformation in vivo (Salzer et al. 2014).

Understanding the function of EpsE and its individual domains is essential for elucidating the mechanism of T2S and its contribution to pathogenesis. In this study, we investigate the role of the tetracysteine motif and zinc in the EpsE C_M_ domain, as EpsE is a well‐characterized ATPase involved in the secretion of cholera toxin and many hydrolytic enzymes in *V. cholerae*, an important human pathogen and established model organism. We show that zinc coordination by the C_M_ domain is required for the function of EpsE in T2S, but the C_M_ residues between the two dicysteines are interchangeable with that of a homolog. Zinc coordination provides stability to the EpsE hexamer, as removal of zinc results in a loss of ATPase activity in vitro, an inability to support T2S in vivo, and an alteration in the protein's conformation.

## Experimental Procedures

### Bacterial strains and growth conditions

*Vibrio cholerae* TRH7000 (El Tor, *thy* Hg^R^ [*ctxA*–*ctxB*]), *V. cholerae* 3083 (El Tor, serotype Ogawa), and *Escherichia coli* BL21(DE3) were used in this study. *Vibrio cholerae* strains were grown at 37°C in LB broth supplemented with 100 mg mL^−1^ thymine for TRH7000. Those strains containing plasmids were grown in the presence of 200 *μ*g mL^−1^ carbenicillin and induced with isopropyl‐d‐thiogalactopyranoside (IPTG) as described in the figure legends.

### Cloning and expression

The EpsE C_M_ domain deletion mutants were constructed using PCR with the following primers: ΔC_M_: 5′‐TAAGGT GCGCACCAAGCGCTGAG‐3′ and 5′‐TACCGTGGCCGA ACCGGTAT‐3′; ΔC_M_Pro: 5′‐CCATACCGTGGCCGAACC GGTAT‐3′. The EpsE ΔC_M_ construct is missing residues 396–437, while ΔC_M_Pro replaces those residues with a proline. The *epsE* fragments containing mutations were cloned into pMMB384 (wild‐type *epsE* in pMMB67EH) (Sandkvist et al. 1995) by the exchange of an MfeI/BamHI fragment to create the pMMB EpsE variant plasmids. The pMMB plasmids were then introduced to *epsE*::*kan* and wild‐type (WT) *V. cholerae* strains through conjugation.

The pMMB*epsE*‐*xcpR* C_M_ chimera plasmid was constructed by first amplifying the beginning of *epsE* and creating a 3′ region of overlap with the beginning of the *xcpR* C_M_ domain (5′‐GAGGGATCCTGAGCAGATGGAAGCCAAGCAATGACCGAA‐3′ [BamHI site underlined] and 5′‐GCGCGGTAGGGCTCCTTGCAATCTGGGCATAAGG‐3′). The *xcpR* C_M_ fragment was amplified from pMMB‐*xcpR* (Turner et al. 1993) using the primers 5′‐CCTTATGCCCAGATTGCAAGGAGCCCTACCGCGC‐3′ and 5′‐TGGTTACATTTAGGGCAGCCGCGGGCGCGATGCA‐3′. The downstream part of *epsE* was then amplified with a 5′ overlap of the end of the *xcpR* C_M_ domain using the primers 5′‐TGCATCGCGCCCGCGGCTGCCCTAAATGTAACCA‐3′ and 5′‐CTCCTGCAGCAAACGCGGCCATTAGGACTCCTTAGTC‐3′ (PstI site underlined). The three amplified fragments were used as a template for the amplification of the entire region using the first and last primers listed (to yield the *epsE*‐*xcpR* C_M_ PCR product) and cloned into pMMB67EH using the BamHI and PstI restriction sites and introduced into *epsE::kan* and WT *V. cholerae* strains through conjugation. The protein purification expression vector pET21(d)*epsE*(2‐503)‐*xcpR*C_M_‐*epsL*(1‐253)‐his_6_ was constructed by exchange of a NotI/BsmI fragment containing the C_M_ domain from the *epsE*‐*xcpR* C_M_ PCR product.

The full‐length EpsE‐Hcp1 fusion is encoded by the expression vector pET21(d)*epsE*(1‐503)‐8aa‐*hcp1*‐his_6_, and the truncated ΔN1‐EpsE‐Hcp1 fusion from pET21(d)*epsE*(100‐503)‐8aa‐*hcp1*‐his_6_. pET21(d)*epsE*(100‐503)‐*xcpR*C_M_‐8aa‐*hcp1*‐his_6_ was created by the exchange of a NotI/BsmI fragment from pET21(d)*epsE*(2‐503)‐*xcpR*C_M_‐*epsL*(1‐253)‐his_6_. The pMMB*epsE*‐*hcp1* and *epsE*‐*xcpR*C_M_‐*hcp1* constructs were made by PCR amplification of the region of interest from pET21(d)*epsE*(100‐503)‐8aa‐*hcp1*‐his_6_ or pET21(d)*epsE*(100‐503)‐*xcpR*C_M_‐8aa‐*hcp1*‐his_6_, respectively, using the primers 5′‐GAGGGATCCGAAGGAGATATACATGGACTTCTTC‐3′ (BamHI site underlined) and 5′‐GAGCTGCAGATATCAGGCCTGCACGTTCTG‐3′ (PstI site underlined) and cloning into pMMB67EH using the BamHI and PstI restriction sites. The plasmids were conjugated into *epsE::kan* and WT *V. cholerae* as before.

EpsE point mutations were constructed using the QuikChange site‐directed mutagenesis kit (Stratagene, Agilent, Santa Clara, CA, USA) as directed. The following primers were used to construct the cysteine mutations: C400S (5′‐CCTTATGCCCAGATTCCAAAGAGCCTTACGAGGC‐3′ and 5′‐GCCTCGTAAGGCTCTTTGGAATCTGGGCATAAGG‐3′), C430S (5′‐CGTGCAACGGGCTCCCCTAAATGTAACC‐3′ and 5′‐GGTTACATTTAGGGGAGCCCGTTGCACG‐3′), C397SC400S (5′‐GGTGCGCACCTTATCCCCAGATTCCAAAGAGCCTTAC‐3′ and 5′‐GTAAGGCTCTTTGGAATCTGGGGATAAGGTGCGCACC‐3′), and C430SC433S (5′‐CG TGCAACGGGCTCCCCTAAATCTAACCACAAAGG‐3′ and 5′‐CCTTTGTGGTTAGATTTAGGGGAGCCCGTTGCACG‐3′). *epsE*C400SC430SC433S (C3xS) was constructed using site‐directed mutagenesis with *epsE*C430SC433S template DNA and the primers for C400S listed above, and *epsE*C397SC400SC430SC433S (C4xS) was made similarly using *epsE*C397SC400S as a template and the primers for C430SC433S. Regions containing mutations were cloned into pMMB384 by exchange of an MfeI/BamHI fragment to create the individual pMMB EpsE variant plasmids. Plasmids were then introduced to *epsE::kan* and WT *V. cholerae* strains through conjugation.

### Protease and lipase secretion assays

Measurements of extracellular protease activity were performed as described previously (Sikora et al. 2007). Briefly, the fluorogenic probe, *n*‐tert‐butoxy‐carbonyl‐Gln‐Ala‐Arg‐7‐amido‐4‐methylcoumarin (Sigma‐Aldrich, St. Lois, MO, USA) was added to overnight culture supernatants and 10‐min kinetic protease activity was measured using fluorescence at excitation and emission wavelengths 385 nm and 440 nm, respectively. Lipase activity was quantified as described previously by incubating *V. cholerae* overnight culture supernatants with 4‐nitrophenyl myristate and measuring 4‐nitrophenol release as the change in absorbance at 415 nm over a 30‐min period (Johnson et al. 2015). All assays were performed in triplicate, normalized to the density of the culture at 600 nm, and mean and SEM are displayed.

### SDS‐PAGE and immunoblotting

Cell lysates were boiled in SDS sample buffer and analyzed by SDS‐PAGE using 4–12% *bis*‐Tris gels (NuPAGE, Invitrogen, Waltham, MA, USA) and MES or MOPS running buffer. Proteins were transferred to nitrocellulose membranes (Protran; GE Healthcare, Buckinghamshire, UK) using NuPage transfer buffer (Invitrogen) and probed with either 1:10,000 *α*‐EpsE antibodies or 1:5000 *α*‐cholera toxin antibodies followed by 1:20,000 horseradish peroxidase‐conjugated goat *α*‐rabbit immunoglobulin G (BioRad) or 1:1,000 *α*‐Hcp1 antibodies followed by 1:10,000 goat *α*‐rabbit IgG‐HRP. Blots were developed using Ecl2 (Pierce, part of ThermoFisher, Waltham, MA, USA) and imaged using a Typhoon FLA 9500 (GE Healthcare).

### Protein purification

Constructs for purification were introduced into pET21(d)*epsE*(100‐503)‐8aa‐*hcp1*‐his_6_ (ΔN1‐EpsE‐Hcp1) (Lu et al. 2013) or pET21(d)*epsE*(1‐503)‐8aa‐*hcp1*‐his_6_ (full‐EpsE‐Hcp1) and expressed in *E. coli* BL21(DE3) under IPTG‐inducing conditions. Proteins were purified using metal‐affinity chromatography on cobalt resin (Talon, Clontech, Mountain View, CA, USA). Subsequently, size‐exclusion chromatography was performed using a Superose 6 column (GE Healthcare) as described previously (Robien et al. 2003; Lu et al. 2013) and compared to known protein standards. Gel filtration fractions containing protein peaks were analyzed using SDS‐PAGE and visualized by staining the gel with Gel Code Blue (Thermo Scientific, Waltham, MA, USA).

### ATPase activity assays

Purified EpsE‐Hcp1 and ΔN1‐EpsE‐Hcp1 fusion proteins were assayed for in vitro ATPase activity according to Lu et al. (2013) using BIOMOL Green reagent (Enzo Life Sciences, Farmingdale, NY, USA) to detect free Pi.

### PAR/PCMB assay

Zinc release was measured using a modified PAR/PCMB assay (Camberg and Sandkvist 2005; Ilbert et al. 2007). Briefly, zinc was removed from ΔN1‐EpsE‐Hcp1 purified protein using a titration of *p*‐chloromercuribenzoic acid (PCMB; Sigma‐Aldrich, St. Lois, MO, USA) in the presence of the zinc‐complexing agent 4‐(2‐pyridylazo)resorcinol (PAR; Sigma, Aldrich). Zinc release was measured at 500 nm and was compared to a ZnCl_2_ standard curve. Assays were performed in duplicate and SEM is shown.

### Native‐PAGE

Proteins were treated with a fourfold molar excess of PCMB or mock treated for 10 min at room temperature and analyzed on a 4–20% Tris‐glycine native gel (NuPAGE, Invitrogen) with Tris‐glycine running buffer at 125 V for 5 h on ice. Proteins were visualized by staining the gel with Gel Code Blue (Thermo Scientific).

### *β*‐Lactamase activity assay

Periplasmic contents were isolated by incubating cells from overnight cultures with 2000 U/mL polymyxin B sulfate in PBS on ice for 30 min, centrifuging at 6.000 × g for 10 min, and isolating the supernatant (periplasmic extract) from spheroplasts. *β*‐Lactamase activity was measured in overnight culture supernatants and periplasmic extracts as previously described with some modifications (Sikora et al. 2007). Nitrocefin (EMD Chemicals, Darmstadt, Germany) was added to supernatants and periplasmic extracts in PBS buffer and the absorbance at 482 nm was measured over the course of 5 min at 37°C.

## Results

### The EpsE C_M_ domain is required for type II secretion

**Figure 2:**
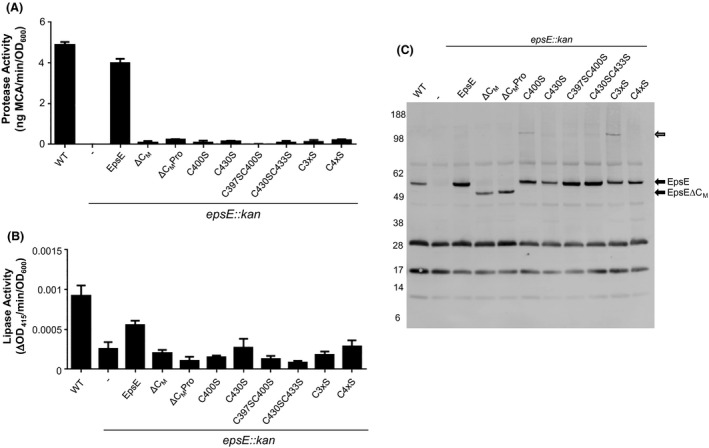
The EpsE C_M_ domain is required for secretion. (A) WT and *epsE::kan* strains of *Vibrio cholerae* TRH7000 containing empty vector (−) or pMMB plasmids encoding WT or mutant EpsE variants described on the *x*‐axis were grown with 200 *μ*g mL^−1^ carbenicillin and 10 *μ*mol L^−1^ IPTG. Culture supernatants were analyzed for VesB protease activity using a cleavable fluorogenic probe as described in Experimental Procedures. All EpsE variants showed statistical significance compared to WT EpsE (*P* < 0.0001). (B) The same overnight culture supernatants as in A were analyzed for lipase activity as described in Experimental Procedures. All EpsE variants showed statistical significance compared to WT EpsE (*P* < 0.03). (C) Expression of EpsE in WT *V. cholerae* TRH7000, followed by *epsE::kan V. cholerae* containing empty vector and *epsE::kan V. cholerae* expressing either WT EpsE or variants of EpsE. Samples were analyzed by SDS‐PAGE and immunoblotting for EpsE. The size of EpsE and EpsE ΔC_M_ are indicated by black arrows, and EpsE dimers by a gray arrow. WT, wild type strain.

**Figure 3:**
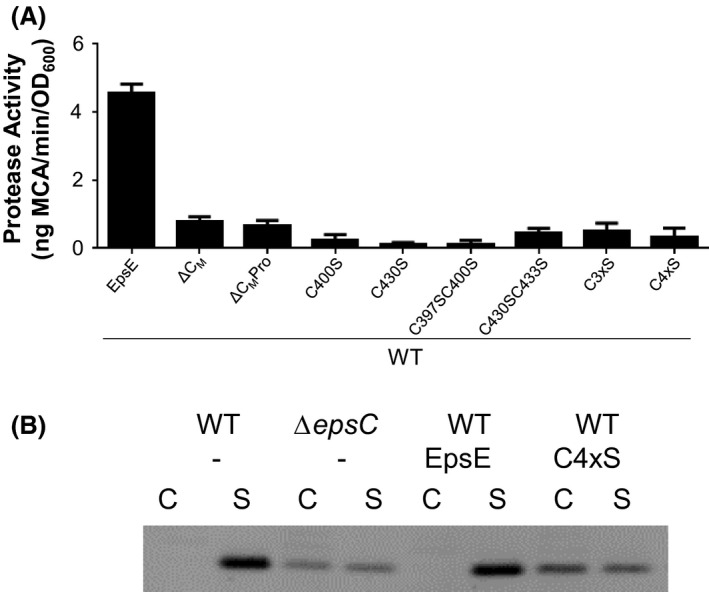
(A) Wild‐type (WT) *Vibrio cholerae* TRH7000 containing pMMB plasmids encoding WT or mutant EpsE variants described on the *x*‐axis were grown with 200 *μ*g mL^−1^ carbenicillin and 100 *μ*mol L^−1^ IPTG. Samples were prepared and assayed for protease activity as in Figure 2. Assays were performed in triplicate and standard error is shown. All EpsE variants showed statistical significance compared to WT EpsE (*P* < 0.0001). (B) WT *V. cholerae* 3083 and isogenic Δ*epsC* containing pMMB plasmids (empty vector [−], WT EpsE, or EpsE C4xS) were grown with 200 *μ*g mL^−1^ carbenicillin and 100 *μ*mol L^−1^ IPTG. Cells (C) and supernatants (S) were analyzed by SDS‐PAGE and immunoblotting for cholera toxin B subunit.

The C_M_ domain is conserved among T2S and T4P assembly ATPases such as EpsE and PilB, while it is absent in homologous T4P retraction ATPases such as PilT and type IV secretion ATPases including HP0525 (Fig. 1), suggesting that it may be required for a process unique to T2S and T4P assembly. In order to examine the importance of the C_M_ domain in T2S, we designed mutations in EpsE that remove the entire C_M_ domain and connect the residues at the point in the CTD where the *β*‐strands most closely converge based on structural superposition of EpsE and HP0525, which lacks the C_M_ domain (Fig. 1). We removed the entire domain including the four cysteines (EpsE ΔC_M_) or replaced it with a proline residue (EpsE ΔC_M_Pro) in order to generate EpsE variants that resemble ATPases lacking the C_M_ domain. Although WT EpsE can complement the loss of secretion of the serine protease VesB and lipase in an *epsE*::*kan* strain of *V. cholerae*, EpsE ΔC_M_ and EpsE ΔC_M_Pro are deficient in their ability to support secretion in this mutant (Fig. 2A, B). While EpsE ΔC_M_ and EpsE ΔC_M_Pro are expressed, they are detected at slightly lower levels than WT EpsE, suggesting a small decrease in stability (Fig. 2C). However, both EpsE ΔC_M_ and EpsE ΔC_M_Pro exhibit negative dominance, as overexpression of these EpsE variants prevents T2S in WT *V. cholerae* (Fig. 3A). This dominant negative phenotype may be explained by competition between WT and mutant forms of EpsE for interaction with other components within the T2S complex, or by the formation of mixed EpsE oligomers with lower overall activity. Together, these results suggest that both deletion constructs are expressed in *V. cholerae*, and that the C_M_ domain is necessary for EpsE's function in T2S.

We have previously reported that *V. cholerae* T2S mutants leak periplasmic content likely due to outer membrane damage (Sikora et al. 2007). Therefore, we also evaluated the ability of EpsE ΔC_M_ and EpsE ΔC_M_Pro to restore outer membrane integrity by determining their effect on nonspecific extracellular release of periplasmic *β*‐lactamase. We observed higher percentages of *β*‐lactamase activity in the supernatant of *epsE*::*kan* strains containing empty vector or expressing the C_M_ deletion variants of EpsE (35–45%) compared to WT *V. cholerae* and the *epsE*::*kan* strain expressing WT EpsE (15–20%) further indicating that these EpsE variants are nonfunctional (Fig. S1).

### Residues in the C_M_ loop are interchangeable for EpsE's function in vivo and in vitro

**Figure 4:**
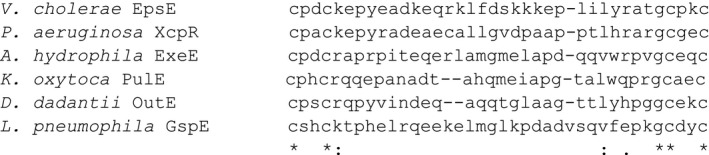
Alignment of C_M_ domains in T2S ATPase homologs. Clustal Omega alignment of CXXCX_27–30_CXXC motifs of T2S ATPase homologs. Asterisks below indicate residue conservation identity, and colons and periods indicate high and low levels of residue homology, respectively.

**Figure 5:**
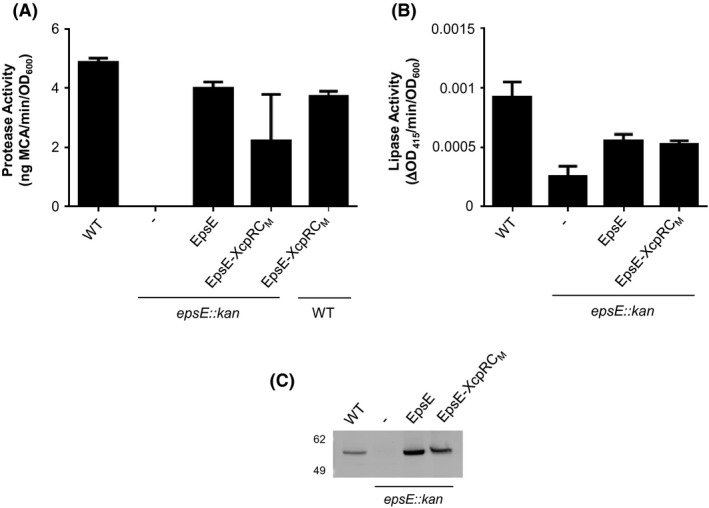
The EpsE‐XcpR C_M_ chimera partially complements the T2S defect in *epsE::kan* mutants of *Vibrio cholerae*. (A) *Vibrio cholerae* TRH7000 WT, followed by *epsE::kan* strains containing empty vector (−) or pMMB encoding EpsE or EpsE‐XcpR C_M_ were grown with 200 *μ*g mL^−1^ carbenicillin and 10 *μ*mol L^−1^ IPTG. The last bar represents WT *V. cholerae* expressing EpsE‐XcpR C_M_ induced with 100 *μ*mol L^−1^ IPTG to test for negative dominance. Culture supernatants were analyzed for VesB activity using a cleavable fluorogenic probe as described in Experimental Procedures. Assays were performed in triplicate and SEM is shown. No statistically significant difference between WT EpsE and EpsE‐XcpR C_M_ was detected using a *t* test (*P* = 0.37). (B) Overnight culture supernatants were assayed for lipase activity as in Figure 2B. No statistically significant difference between WT EpsE and EpsE‐XcpR C_M_ was detected using a *t* test (*P* = 0.064). (C) Expression of EpsE in WT *V. cholerae* TRH7000 (lane 1), followed by empty vector (lane 2), WT EpsE (lane 3), or EpsE‐XcpR C_M_ expressed in *epsE::kan V. cholerae* (lane 4) and induced with 10 *μ*mol L^−1^ IPTG. Samples were analyzed by SDS‐PAGE and immunoblotting for EpsE.

After establishing the importance of the EpsE C_M_ domain, we then investigated the role of the 29 amino acids in between the two dicysteines. In order to understand the contribution of these residues to EpsE's function, we decided to swap the loop from EpsE with that of a homolog. This technique was selected over mutation of individual residues as it allowed us to simultaneously substitute multiple residues and to evaluate whether the C_M_ loop residues participate in species‐specific protein–protein interactions (Sandkvist et al. 1995, 2000). We compared the C_M_ loop residues among several EpsE homologs and found that both the length of the loop and the specific residues vary significantly (Fig. 4). The EpsE C_M_ loop contains 29 amino acids, and in order to alter the specific residues of the C_M_ loop without changing the length, we chose to genetically engineer a chimeric construct, EpsE‐XcpR C_M_, wherein the 29 C_M_ loop residues between the two dicysteines from EpsE were exchanged with those of XcpR from *Pseudomonas aeruginosa*. The cysteines remain intact, but the exchanged region from EpsE differs from that of XcpR by 17 out of 29 residues, mostly in the central portion of the loop (Fig. 4). The EpsE‐XcpR C_M_ chimera was found to complement the loss of VesB and lipase secretion in the *epsE*::*kan* strain of *V. cholerae* (Fig. 5A, B) and is expressed at nearly WT levels (Fig. 5C). Although restoration of protease secretion was much more variable compared to WT EpsE, it was consistently more substantial than the C_M_ deletion mutant phenotypes (Fig. 2A, B). Consistent with its ability to complement the secretion defect in the *epsE*::*kan* mutant, the EpsE‐XcpR C_M_ chimera had no negative effect on VesB secretion when overexpressed in WT *V. cholerae* (Fig. 5A). It is possible that either the presence of WT EpsE can overcome a slight defect in the interaction of the EpsE‐XcpR C_M_ chimera with the T2S system or that this chimera causes a deficiency in oligomer formation or stability.

**Figure 6:**
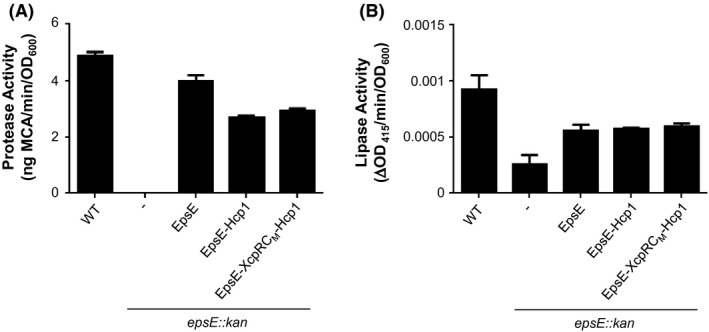
EpsE‐Hcp1 and EpsE‐XcpR C_M_–Hcp1 fusions support secretion in *Vibrio cholerae*. (A) WT and *epsE::kan* strains of *V. cholerae* TRH7000 containing empty vector (−), pMMB encoding EpsE, EpsE‐Hcp1, or EpsE‐XcpR C_M_–Hcp1 were grown with 200 *μ*g mL^−1^ carbenicillin and 10 *μ*mol L^−1^ IPTG. Supernatants were analyzed for VesB activity using a cleavable fluorogenic probe as described in Experimental Procedures. Assays were performed in triplicate and standard error is shown. (B) Lipase assays were performed on overnight culture supernatants as in Figure 2B. Assays were performed in triplicate with standard errors displayed.

**Figure 7:**
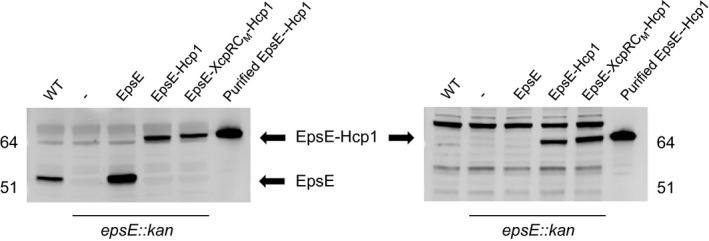
Detection of full‐length EpsE‐Hcp1 fusions in *Vibrio cholerae*. Overnight cultures of *V. cholerae* TRH7000 WT or *epsE*::kan strains containing empty vector (−) or pMMB plasmids encoding EpsE, EpsE‐Hcp1, or EpsE‐XcpR C_M_–Hcp1 were grown with 200 *μ*g mL^−1^ carbenicillin and 10 *μ*mol L^−1^ IPTG. Cell lysates were analyzed by SDS‐PAGE and immunoblotting using *α*‐EpsE antibodies (left) or *α*‐Hcp1 antibodies (right). Molecular mass markers are indicated and the positions of EpsE and EpsE‐Hcp1 fusions are shown with arrows. Purified EpsE‐Hcp1 protein was included as a positive control.

**Figure 8:**
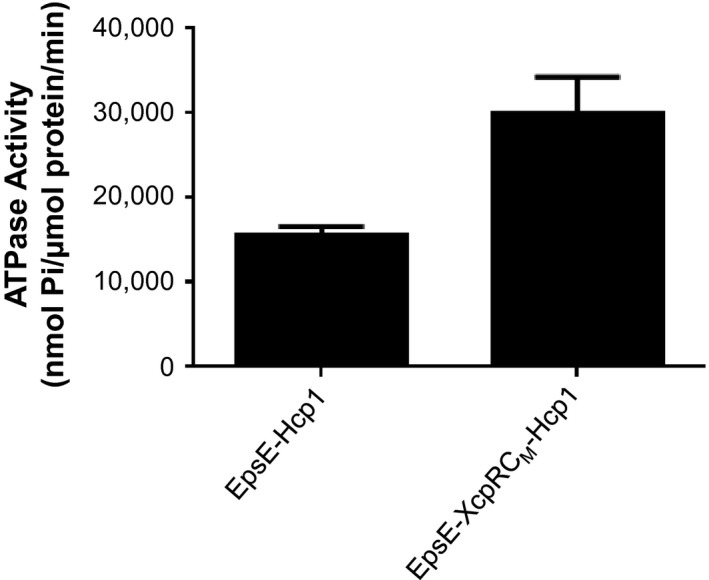
The EpsE‐XcpR C_M_–Hcp1 chimera fusion maintains in vitro ATPase activity. Purified EpsE‐Hcp1 and EpsE‐XcpR C_M_‐Hcp1 fusions were assayed for in vitro ATPase activity as described in Experimental Procedures.

EpsE hexamers have a greatly increased ATPase activity over monomeric EpsE, suggesting that EpsE likely functions as a hexamer in vivo (Camberg and Sandkvist 2005). The crystal structure of EpsE was first solved as a helical filament, but modeled as a hexamer based on the structure of *Helicobacter pylori* HP0525 (Yeo et al. 2000; Robien et al. 2003). In a later study, a refined hexameric model was proposed based on *P. aeruginosa* PilT and tested by mutagenesis of residues predicted to participate in subunit–subunit interactions (Patrick et al. 2011). We recently constructed a fusion protein consisting of Hcp1, a hexameric protein from *P. aeruginosa* (Mougous et al. 2006), fused to the C‐terminus of EpsE (Lu et al. 2013). This resulted in a stable EpsE hexamer with increased ATPase activity that was amenable to purification, crystallization, and structure determination. Similarly, the C‐terminal Hcp1 fusion strategy has also been recently used to purify the hexameric form of the homologous T4P assembly ATPase PilB (Bischof et al. 2016). To determine whether stabilization of the EpsE‐XcpR C_M_ chimera by the “assistant hexamer” Hcp1 can overcome a possible oligomerization defect, we fused Hcp1 to EpsE‐XcpR C_M_ and compared its ability to support secretion with EpsE‐Hcp1 in the *epsE*::*kan* mutant. We found that the EpsE‐XcpR C_M_–Hcp1 chimera fusion with the loop swap is able to support T2S to the same extent as EpsE‐Hcp1 (Fig. 6), suggesting that most of the residues in the EpsE C_M_ domain can be interchanged with those of a homolog without having a negative impact on EpsE's ability to support T2S. In order to verify that the fusions are stable and do not break down into native EpsE, we analyzed cell extracts by SDS‐PAGE and immunoblotting for EpsE and Hcp1 (Fig. 7). The results show that both WT and chimeric EpsE‐Hcp1 fusions remain intact in vivo and migrate according to their predicted molecular weights similarly to EpsE‐Hcp1‐His_6_ purified by metal‐affinity and size‐exclusion chromatography from *E. coli* cell extracts (Figs. S2, S3). Thus, the complementation of the T2S defect in the *epsE*::*kan* mutant by EpsE‐Hcp1 and EpsE‐XcpR C_M_‐Hcp1 is likely due to the intact fusions and not breakdown products, indicating that the fusions are functional. In addition, these results show that the variability in complementation of T2S by the EpsE‐XcpR C_M_ chimera is mitigated when the assistant hexamer Hcp1 is present, suggesting that there is no apparent species‐specific role of the C_M_ loop residues between the dicysteines. As expected, when we overexpressed and purified hexameric His_6_‐tagged forms of EpsE‐Hcp1 and EpsE‐XcpR C_M_‐Hcp1 from *E. coli* by metal‐affinity chromatography and gel filtration (Figs. S2, S3) and determined their ability to hydrolyze ATP, there is no decrease in ATPase activity when the EpsE C_M_ loop residues are exchanged with those of the homolog XcpR (Figs. 8).

### Zinc binding to the C_M_ domain is required for T2S and EpsE ATPase activity

Given the importance of having an intact C_M_ domain, and yet the relative flexibility in the requirement for precise C_M_ loop residues for the function of EpsE, we next sought to understand the contribution of the dicysteines to EpsE and T2S. We constructed a series of EpsE C_M_ cysteine to serine substitution mutants and tested their ability to complement the loss of T2‐secreted protease and lipase activities and restoration of outer membrane integrity in the *epsE::kan* mutant as described earlier. We analyzed single (C400S, C430S), double (C397SC400S, C430SC433S), triple (C400SC430SC433S), and quadruple (C397SC400SC430SC433S) cysteine to serine substitution mutants. Similarly to the C_M_ domain deletion variants EpsE ΔC_M_ and EpsE ΔC_M_Pro, all EpsE C_M_ cysteine mutants are unable to complement the T2S defects in the *epsE::kan* strain (Figs. 2 and S1). While expression of the C400S and C3xS variants results in what appear to be EpsE dimers, perhaps due to the exposure of single free cysteines, these bands are not detected in the double or quadruple cysteine mutants (Fig. 2C). In order to test whether the cysteine mutant variants of EpsE are nonfunctional simply due to misfolding, we tested for negative dominance in vivo. All cysteine mutants exhibited negative dominance and inhibited the ability of WT EpsE to support protease secretion, suggesting that they are not completely misfolded and are able to interact with WT EpsE and/or other components of the T2S complex such as EpsL (Fig. 3A). We also confirmed the negative dominant phenotype of the EpsE C4xS variant by analyzing cholera toxin secretion, and show that expression of EpsE C4xS inhibits the secretion of the cholera toxin B subunit in WT *V. cholerae* to approximately the same level as in a T2S mutant (Fig. 3B). Collectively, these results indicate the importance of cysteines for EpsE's function in T2S and suggest that all four cysteine residues are essential.

In order to analyze the metal content of the cysteine mutant proteins and the effect of these mutations on activity, we attempted to purify hexahistidine‐tagged proteins; however, these variant proteins were not amenable to purification, as they aggregated when overexpressed in *E. coli*. We were also unable to purify cysteine mutant variants of the EpsE‐Hcp1 fusion (data not shown). This suggests that the folding and/or stability of the mutant proteins is severely compromised when any of the cysteine residues are exchanged for serines, and lends support to the notion that zinc binding to this domain may be crucial for the overall conformation/stability of EpsE.

**Figure 9:**
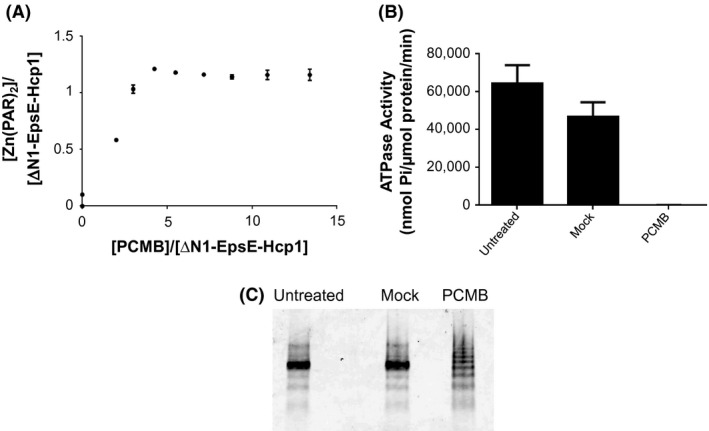
Removal of zinc results in a loss of in vitro ATPase activity and changes the migration pattern of ΔN1‐EpsE‐Hcp1. (A) Zinc release titration curve. Increasing amounts of *p*‐chloromercuribenzoic acid (PCMB) result in increased zinc release. (B) Treatment of ΔN1‐EpsE‐Hcp1 protein abolishes in vitro ATPase activity. Proteins were either untreated or treated with a fourfold molar excess of PCMB or mock treated for 10 min at room temperature and assayed for ATPase activity as described in Experimental Procedures. (C) Purified ΔN1‐EpsE‐Hcp1 was untreated or incubated with a fourfold molar excess of PCMB or mock treated for 10 min at room temperature. Samples were then analyzed using native‐PAGE and stained with Coomassie.

Because we were unable to analyze the effect of the cysteine‐to‐serine substitutions on purified proteins, we instead took a chemical approach to determine the contribution of zinc binding to the C_M_ domain. We used PCMB to release zinc and measured free zinc using the zinc‐complexing agent 4‐(2‐pyridylazo)resorcinol (PAR). As these experiments required large amounts of purified proteins, we used the previously described hexameric N‐terminally truncated ΔN1‐EpsE‐Hcp1 fusion (Lu et al. 2013), which can be purified in much greater quantities and has about threefold higher in vitro ATPase activity than the full‐length fusion (Figs. S4, Fig. 9B). This form of EpsE is unable to function in vivo because it lacks the first 90 NTD residues known to interact with EpsL, which are necessary for EpsE to support T2S (Sandkvist et al. 1995, 2000; Abendroth et al. 2005) (data not shown). Figure 9A shows that increasing amounts of PCMB cause an increase in the amount of zinc released by ΔN1‐EpsE‐Hcp1 hexamers, which coordinate metal at a 1:1 ratio of zinc:EpsE, consistent with our previous findings from monomeric EpsE (Camberg and Sandkvist 2005). Additionally, when the EpsE‐Hcp1 fusion is pretreated with a fourfold molar excess of PCMB for 10 min at room temperature, it has nearly abolished ATPase activity in vitro compared to untreated and mock‐treated controls (Fig. 9B). Thus, zinc binding to the EpsE C_M_ domain is necessary for in vitro ATPase activity.

### Zinc stabilizes the conformation of EpsE

Based on the aggregation of EpsE C_M_ cysteine mutants when overexpressed in *E. coli* and the loss of ΔN1‐EpsE‐Hcp1's in vitro ATPase activity upon zinc release, we hypothesized that zinc contributes to the overall protein conformation of hexameric EpsE. In order to test this, we analyzed purified protein migration profiles using native‐PAGE. Purified ΔN1‐EpsE‐Hcp1 protein was either untreated or incubated with a fourfold molar excess of PCMB or mock treated for 10 min at room temperature. As seen in Figure 9C, zinc removal results in a change in the native migration pattern of the ΔN1‐EpsE‐Hcp1 hexamer, indicating that conformational changes occur following zinc release.

## Discussion

This study demonstrates that the EpsE C_M_ domain is required for type II secretion in *V. cholerae*. Although many of the loop residues that form the elbow of the C_M_ domain in between the dicysteines are interchangeable, the tetracysteine motif (CXXCX_29_CXXC) must be intact in order for EpsE to function as the molecular motor for T2S. Substitution of any of the cysteine residues resulted in EpsE variants that are unable to support secretion. These EpsE variants are produced in *V. cholerae*; however, none of them were amenable to purification due to aggregation when overexpressed in *E. coli*. It is possible that these variant proteins are misfolded when they are overproduced in isolation from the rest of the T2S complex. However, the negative dominance by the EpsE cysteine mutants when expressed in WT *V. cholerae* demonstrates that they are still able to interact with other components of the T2S complex and/or WT EpsE, suggesting that they retain some native properties and are not completely misfolded. For several of our experiments, we utilized a form of EpsE fused to the assistant hexamer Hcp1, as this construct forms hexamers in the absence of other Eps proteins, has high ATPase activity in vitro (Figs. S2–S4 and Fig. 8) (Lu et al. 2013) and is functional in vivo (Fig. 6). When zinc was chemically removed from purified ΔN1‐EpsE‐Hcp1 fusion protein using PCMB, we observed a loss of ATPase activity and a change in the migration pattern using native‐PAGE (Fig. 9). Collectively, these data indicate that zinc binding to the C_M_ domain is necessary to support hexameric complex stability.

We have previously characterized many aspects of the function and activity of EpsE. Among others, we have shown that while monomeric EpsE is capable of hydrolyzing ATP, hexamerization results in greatly increased ATPase activity (Camberg and Sandkvist 2005; Camberg et al. 2007; Patrick et al. 2011; Lu et al. 2013). The role of zinc was first examined using purified GST‐tagged EpsE that primarily yields monomers with low ATPase activity and only a small fraction of highly active hexamers (Camberg and Sandkvist 2005). Titration of this purified material with *p*‐hydroxymercuriphenylsulfonic acid (PMPS) in the presence of PAR revealed that EpsE binds 1 mol of zinc per mol of EpsE, which corresponds with our results in the present study showing that equimolar amounts of zinc are released by ΔN1‐hexameric EpsE‐Hcp1 using PCMB. When purified EpsE was treated with a fourfold molar excess of PMPS, there was only a 50% decrease in EpsE's ATPase activity, suggesting that zinc does not significantly contribute to the stability of monomeric EpsE (Camberg and Sandkvist 2005). In contrast, our current study shows that there is a nearly complete reduction in ATPase activity of EpsE hexamers. As the ability to hydrolyze ATP is sensitive to the conformational state of EpsE, the removal of zinc has a greater impact on the ATPase activity of the hexamer than the monomer.

Previous studies examining the tetracysteine motifs of similar type II/IV secretion ATPases have also indicated the importance of this motif, although some key differences also exist between those reports and the current study. Possot and Pugsley (1997) showed that secretion of the T2S substrate pullulanase was decreased upon substitution of single or double cysteine to serine substitutions to the ATPase PulE, and was abrogated following a triple cysteine to serine substitution. The two different single cysteine substitutions also exhibited varying amounts of negative dominance, with C391S exhibiting 31% dominance and C419S only 3% (Possot and Pugsley 1997). We have found that even removing a single cysteine abolishes the ability of EpsE to support T2S, and all variants display very similar levels of negative dominance. Unlike WT PulE, which cannot be purified due to the aggregation of the protein when produced in the absence of other T2S components, WT EpsE is soluble and readily amenable to purification. This allowed us to determine the difference in protein solubility upon substituting cysteine residues, which showed that all cysteines are required for EpsE solubility when overexpressed in *E. coli*.

An investigation of the tetracysteine motif in *T. thermophilus* PilF by Salzer et al. (2014) showed these cysteines are required in order to support piliation at high temperatures. PilF requires at least three cysteine residues to coordinate zinc, and may be able to use H_2_O to substitute for the fourth cysteine. These authors used cysteine to alanine substitutions, whereas we chose to substitute cysteines with serines in order to maintain more closely related amino acid side chains. At lower growth temperatures, however, cysteine substitutions do not affect PilF function, suggesting that zinc binding is not essential for pilus assembly, but rather provides the protein stability necessary for proper function at elevated temperatures. Our data indicate that EpsE's stability is also compromised when zinc is removed; however, EpsE is nonfunctional upon replacement of even one of its cysteines. Additionally, zinc is not necessary for PilF ATPase activity, whereas our data show that zinc is required for ATPase activity of EpsE. PilF requires neither ATP nor zinc‐coordinating cysteine residues for hexameric complex assembly and hexamerization does not appear to be a prerequisite for ATP hydrolysis, perhaps due to its extended N‐terminus, which the authors suggest may provide additional stability compared to homologs lacking these additional residues. As this study focused on an extremophile, our results are more likely to be widely applicable to other mesophilic organisms, including important pathogens that express T2S and/or T4P systems.

Zinc frequently plays an important role in protein conformation and stabilization, with zinc‐coordinating domains most commonly supporting overall or domain‐specific protein folding and/or stability or participating in interactions with DNA, RNA, or proteins (Krishna et al. 2003; Maret and Li 2009). Zinc may stabilize a particular conformation that is important for activity, such as a redox sensor, or to position a domain for protein–protein interactions. For example, the chaperone Hsp33 acts as a molecular “redox switch,” by remaining inactive in its zinc‐coordinating reduced state and becoming activated upon cellular oxidation, resulting in disulfide bonding and dimerization (Jakob et al. 1999, 2000; Graumann et al. 2001). SecA, on the other hand, contains a zinc‐binding domain that stabilizes the position of basic residues involved in SecB interactions. (Fekkes et al. 1999; Zhou and Xu 2003). Similarly, the ATP‐dependent chaperone ClpX interacts with the cofactor SspB_2_ via a hydrophobic patch of residues located in a zinc‐binding domain (Thibault et al. 2006).

**Figure 10:**
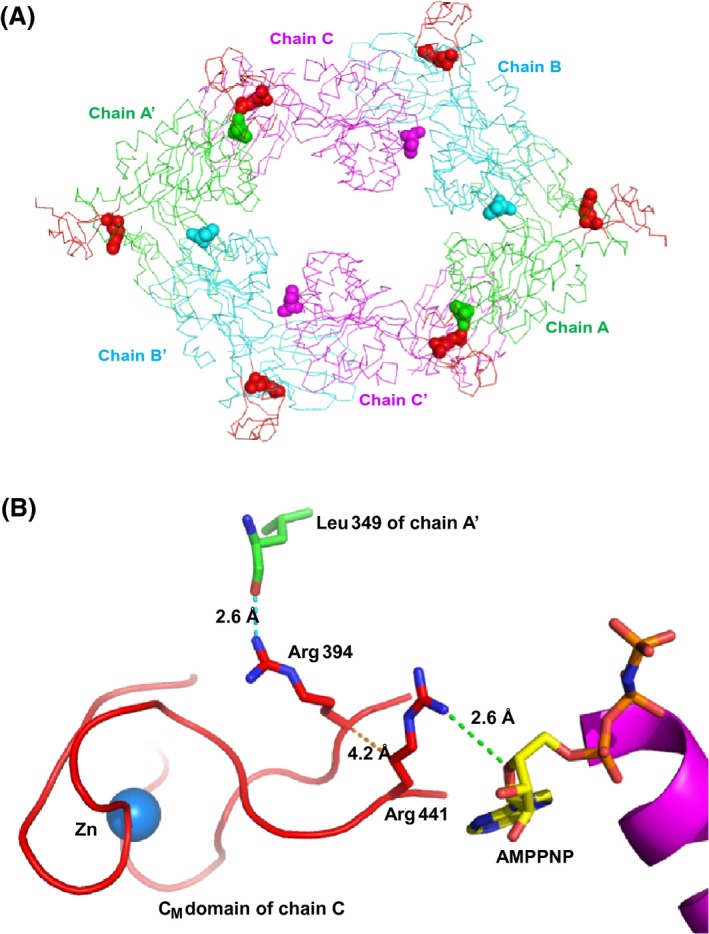
Close‐up view of residues at the base of the C_M_ domain and potential interactions with adjacent subunits or nucleotide. (A) View along the twofold axis of the *Vibrio cholerae* EpsE‐8aa‐Hcp1 hexamer with C2 symmetry (Lu et al. 2013) (PDB code 4KSR). The three independent chains are related by a twofold axis and are displayed as green (chains A, A’), cyan (B, B’), and magenta (C, C’) with C_M_ domains colored red. Arg 394 residues from each subunit are displayed as red spheres, and Leu 349 residues are displayed as spheres according to the color of the corresponding subunit. (B) Arg 394 from chain C is shown in proximity to Leu 349 of chain A’. The proximity of Arg 441 to AMPPNP is also shown, with an alpha‐helix from the EpsE C‐terminal domain in purple. Distances between possible contacts are indicated by dotted lines and labeled. Zinc is superimposed from the structure of monomeric EpsE (Robien et al. 2003) (PDB code 1P9W) and is displayed as a blue sphere.

The work presented here demonstrates that the zinc‐coordinating C_M_ domain is necessary for the activity and function of EpsE, the motor protein that energizes T2S. One lingering question is whether the EpsE C_M_ domain shares any function found in similar zinc‐coordinating domains, such as Hsp33 and SecA. We have not ruled out the possibility that zinc binding to the C_M_ domain may offer a means for bacteria to modulate energy production for T2S in addition to, or as a consequence of, providing protein stability. EpsE is unlikely to act as a redox switch similar to Hsp33, as removal of zinc causes a conformational change resulting in a complete loss of activity. It is feasible that the C_M_ domain is necessary for correctly positioning residues involved in protein–protein interactions in a similar manner to SecA and ClpX; however, if the loop residues participate in protein–protein interactions, this interaction is largely insensitive to the amino acid substitutions in the EpsE‐XcpR C_M_ loop chimera (Fig. 4). Potentially, modulation of the C_M_ domain through C_M_ zinc coordination/abrogation or protein–protein interaction could affect EpsE activity by altering the positioning of important residues in the *β*‐strands that enter and exit the C_M_ domain (Figs. 1, 10). Structural analysis indicates that R441 in the strand leaving the C_M_ domain contacts the adenyl and ribose moieties of the nucleotide (Fig. 10) (Robien et al. 2003). On the opposite strand, entering the C_M_ domain, R394 contacts a leucine residue in a neighboring subunit in two of the six subunits in the elongated hexameric structure of ΔN1‐EpsE‐8aa‐Hcp1 (Fig. 10) (Lu et al. 2013). Changes in the contacts between subunits in the EpsE hexamer might prevent the adoption of important transient conformations that are essential for the functioning of EpsE in T2S. Repositioning of R394 and R441 in Zn‐depleted EpsE variants could explain the loss of ATPase activity in vitro and/or T2S function in vivo.

The T2S system shares many structural similarities not only to the closely related T4P system, but also to the archaeal flagellar system (archaellum) and competence systems of Gram‐positive bacteria (Korotkov et al. 2012). The assembly ATPases supporting each of these systems share many structural features, including C_M_ domains; therefore, results of this study should inform further research not only on T2S, but also among many different molecular motor systems across bacterial and archaeal domains (Planet et al. 2001; Robien et al. 2003; Korotkov et al. 2012). Knowledge about EpsE structure and function relationships may also provide insight into mechanisms of the antagonistic functions of the T4P assembly and retraction ATPases.

## Conflict of Interest

None declared.

## Supporting information


**Figure S1.** Cysteines in EpsE are required for outer membrane stability in *Vibrio cholerae*.
**Figure S2.** Purification of hexameric EpsE‐Hcp1.
**Figure S3.** Purification of hexameric EpsE‐XcpRCM‐Hcp1.
**Figure S4.** Purification of hexameric ΔN1‐EpsE‐Hcp1.
