# Supplementary material for: Zinc coordination is essential for the function and activity of the type II secretion ATPase EpsE
Source: Microbiologyopen. 2016 May 10;5(5):870–82. doi: 10.1002/mbo3.376 (PMC5061722; doi:10.1002/mbo3.376)
Supplement: Supplementary file 1 — Figure S1. Cysteines in EpsE are required for outer membrane stability in Vibrio cholerae. Figure S2. Purification of hexameric EpsE‐Hcp1. Figure S3. Purification of hexameric EpsE‐XcpRCM‐Hcp1. Figure S4. Purification of hexameric ΔN1‐EpsE‐Hcp1. [file MBO3-5-870-s001.pdf]

Zinc Coordination is Essential for the Function and Activity of the Type II Secretion ATPase EpsE

Chelsea S. Rule[1], Marcella Patrick[1$], Jodi L. Camberg[2#], Natalie Maricic[1], Wim G. Hol[3], Maria Sandkvist[1*]

[1]Department of Microbiology and Immunology, University of Michigan Medical School, Ann Arbor, MI
[2]Department of Medicine, Division of Infectious Diseases, University of Maryland School of Medicine, Rockville, MD
[3]Department of Biochemistry, Biomolecular Structure Center, University of Washington, Seattle, WA

*Corresponding author. E-mail address: mariasan@umich.edu, 734-764-3552

SUPPORTING INFORMATION

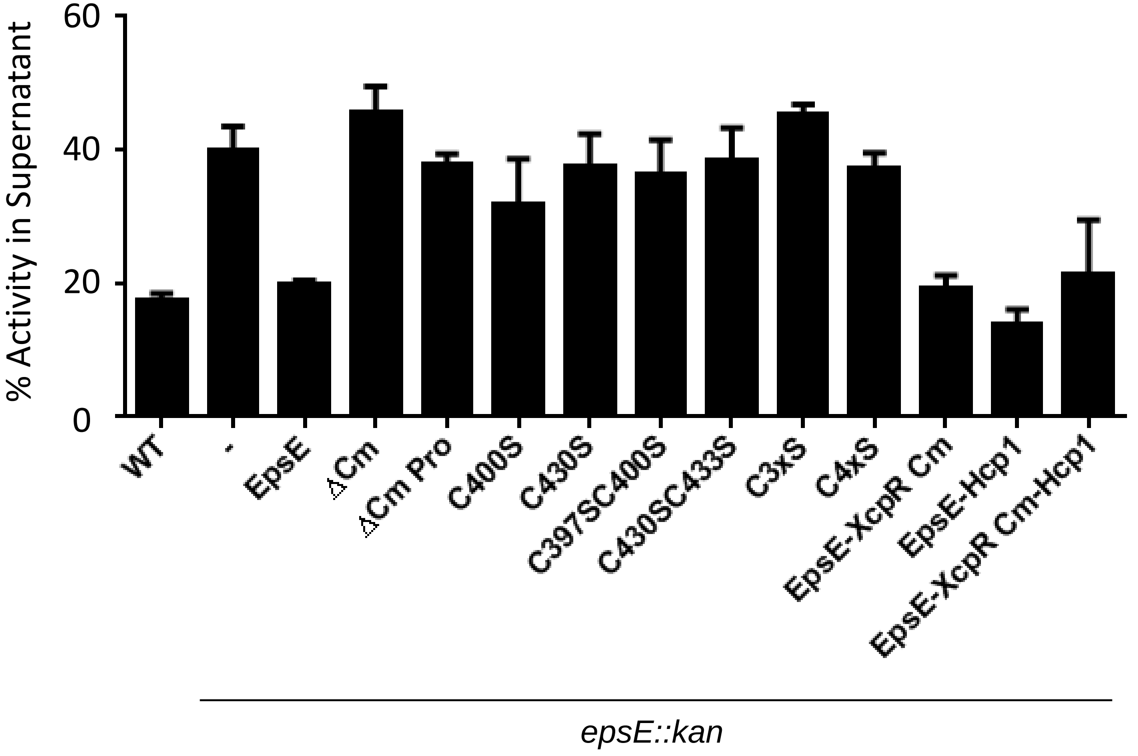

Supplementary Figure 1. Cysteines in EpsE are required for outer membrane stability in *V. cholerae*. Overnight culture supernatants and periplasmic extracts were isolated from WT *V. cholerae* as well as strains of *V. cholerae epsE::kan* containing empty vector or expressing WT and C_M variants of EpsE. β-lactamase activity was measured as described in *Experimental Procedures* and expressed as the percent of total activity in the supernatant. Assays were performed in triplicate, and means and SEM are presented.

A.

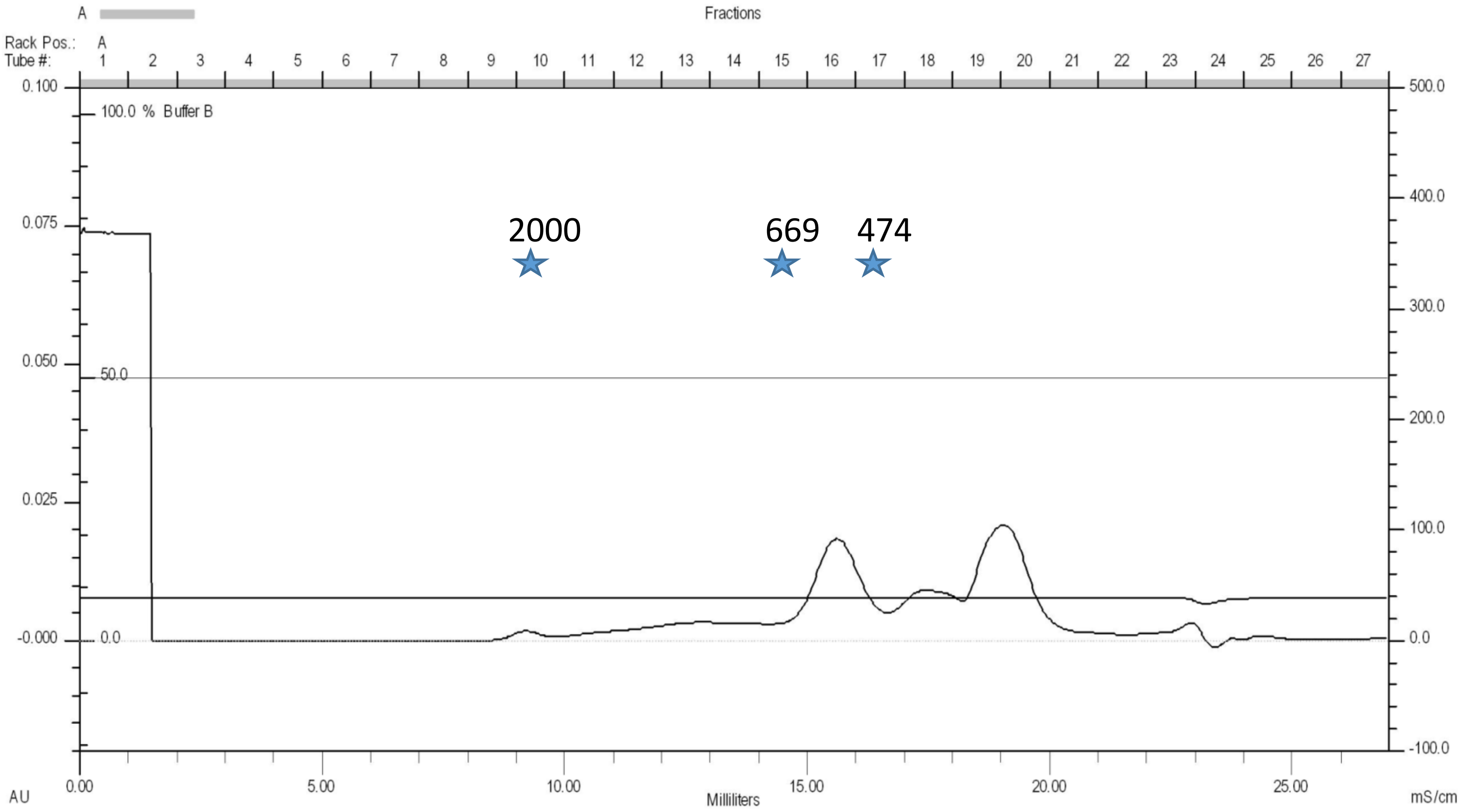

Fractions
Rack Pos.: A
Tube #: 1 2 3 4 5 6 7 8 9 10 11 12 13 14 15 16 17 18 19 20 21 22 23 24 25 26 27
100.0 % Buffer B
50.0
0.0
2000
669
474
0.100
0.075
0.050
0.025
-0.000
500.0
400.0
300.0
200.0
100.0
0.0
-100.0
0.00
5.00
10.00
15.00
20.00
25.00
Milliliters
AU
mS/cm

B.

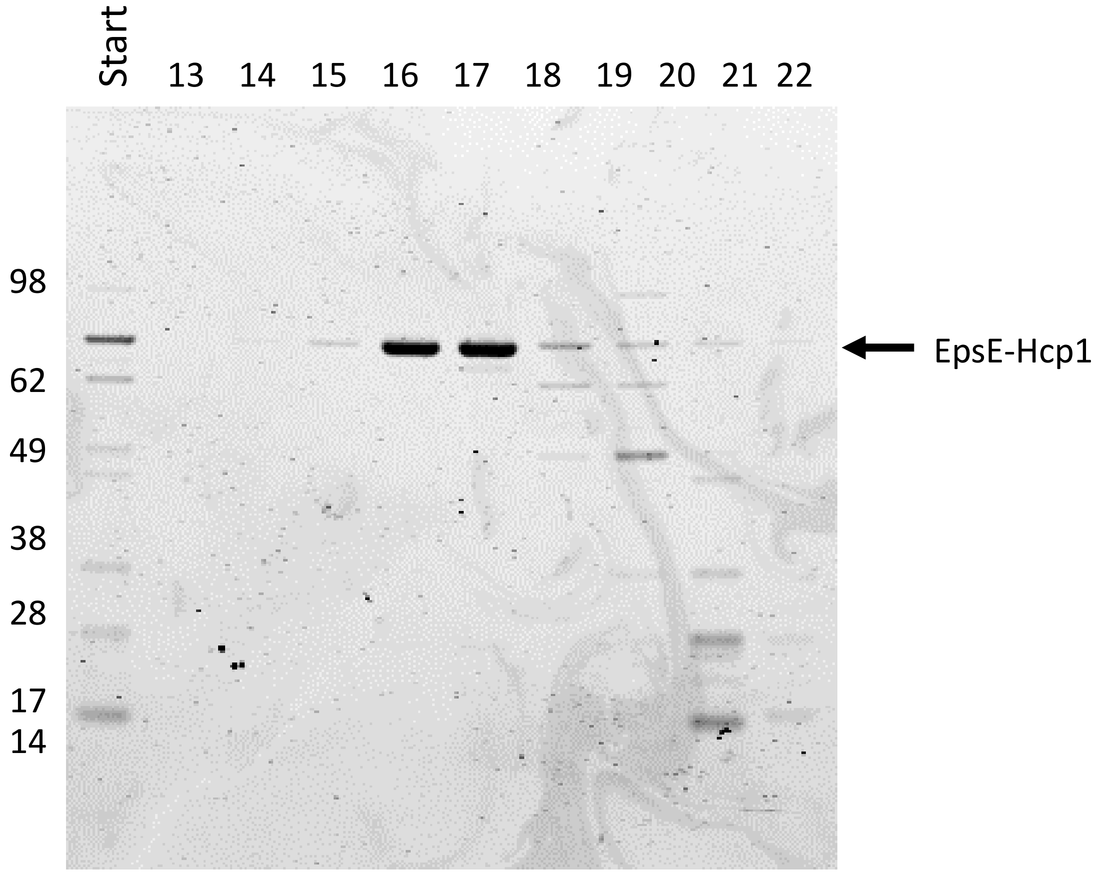

Supplementary Figure 2. Purification of hexameric EpsE-Hcp1. A. EpsE-Hcp1 was purified by metal affinity chromatography and then subjected to gel filtration using a Superose 6 column. Stars represent protein standards with sizes in kDa indicated above. **B**. Fractions containing protein peaks were analyzed via SDS-PAGE and proteins visualized with Coomassie staining. Fractions 16 and 17 were pooled. The size of EpsE-Hcp1 is indicated with an arrow.

A.

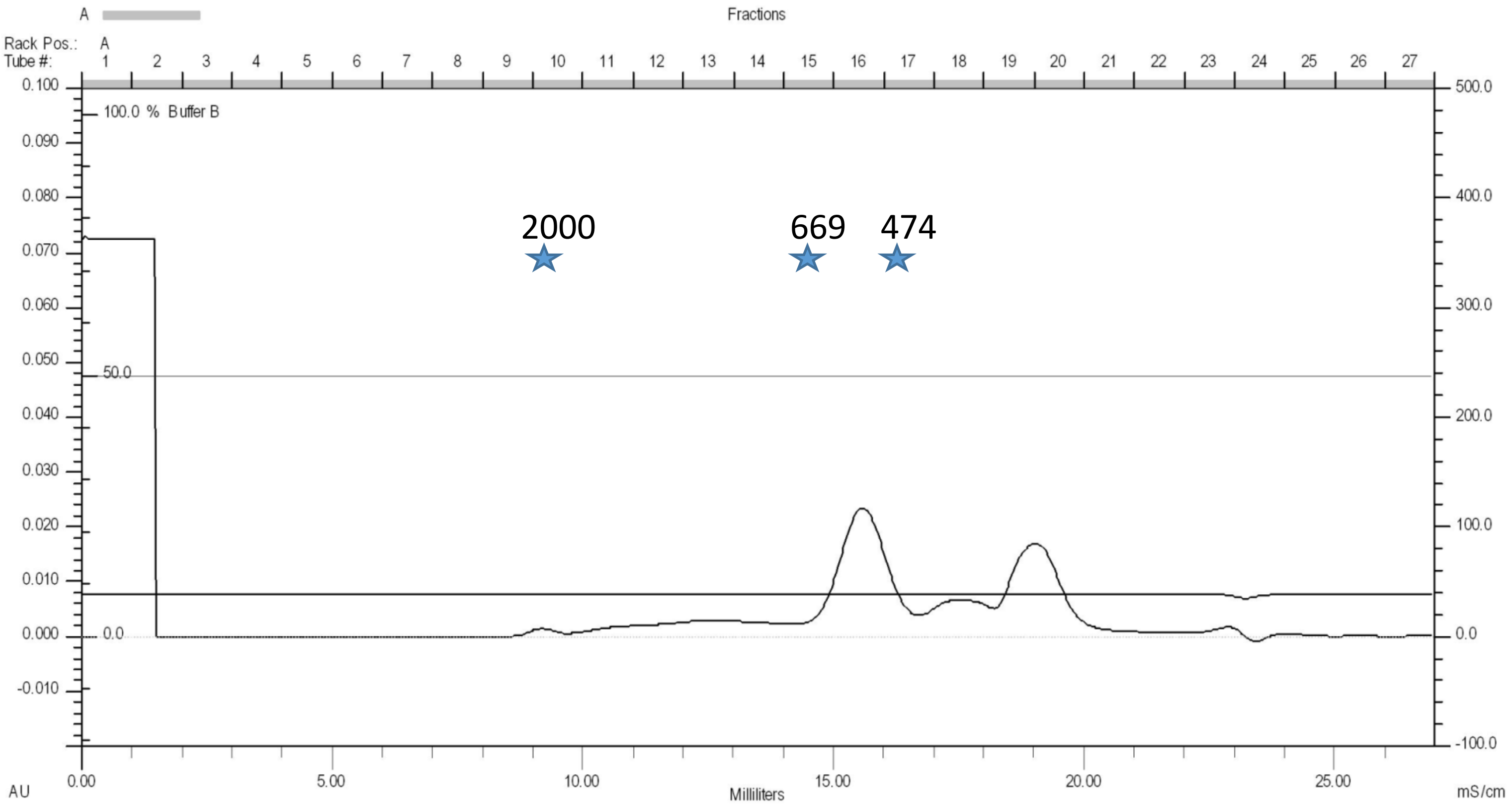
Fractions
Rack Pos.: A
Tube #: 1 2 3 4 5 6 7 8 9 10 11 12 13 14 15 16 17 18 19 20 21 22 23 24 25 26 27
100.0 % Buffer B
50.0
0.0
2000
669
474
0.100
0.090
0.080
0.070
0.060
0.050
0.040
0.030
0.020
0.010
0.000
-0.010
AU
500.0
400.0
300.0
200.0
100.0
0.0
-100.0
mS/cm
0.00
5.00
10.00
15.00
20.00
25.00
Milliliters

B.

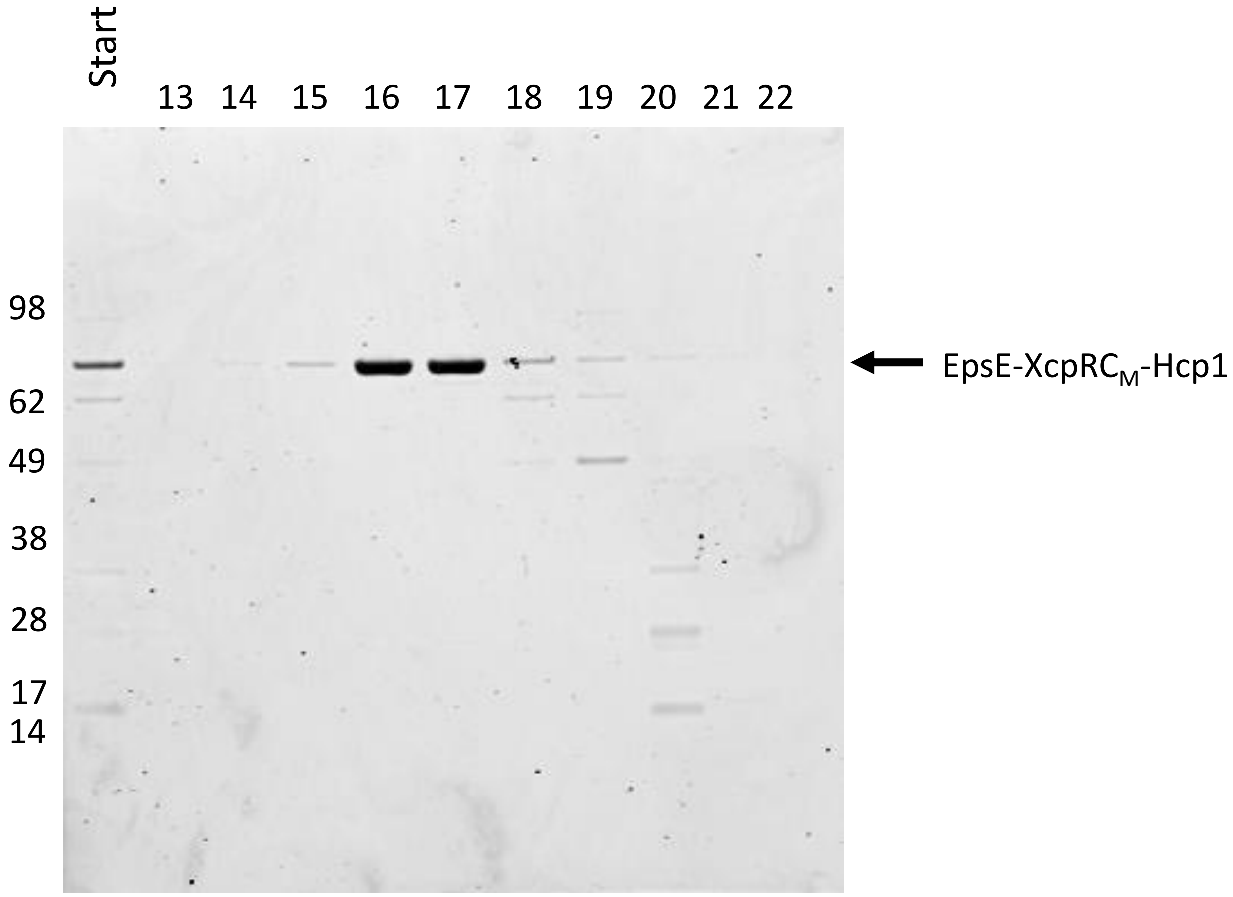

Supplementary Figure 3. Purification of hexameric EpsE-XcpRC$_M$-Hcp1. A. EpsE-XcpRC$_M$-Hcp1 was purified by metal affinity chromatography and then subjected to gel filtration using a Superose 6 column. Stars represent protein standards with sizes in kDa indicated above. **B**. Fractions containing protein peaks were analyzed via SDS-PAGE and proteins visualized with Coomassie staining. Fractions 16 and 17 were pooled. The size of EpsE-XcpRC$_M$-Hcp1 is indicated with an arrow.

A.

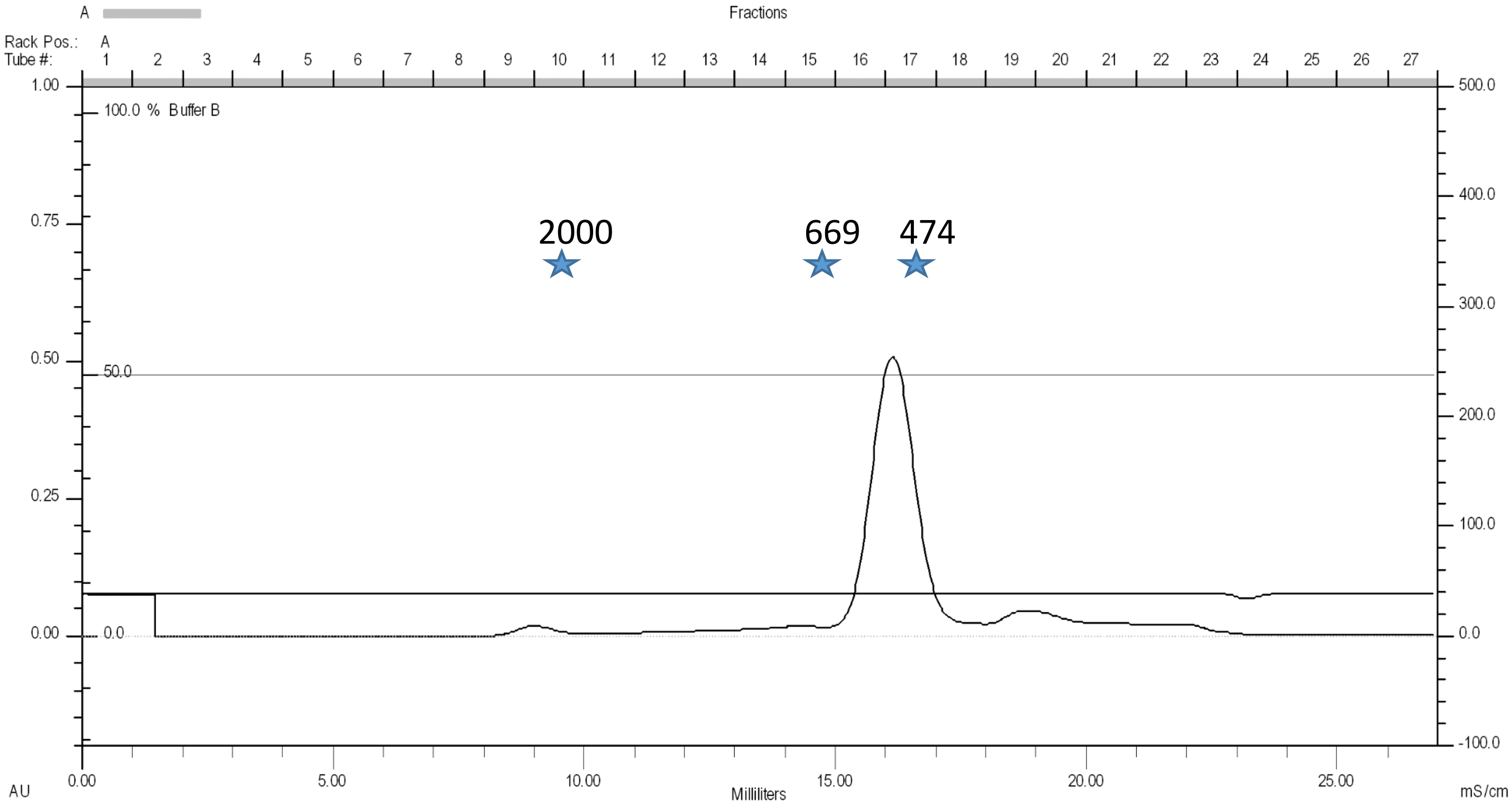

A
Fractions
Rack Pos.: A
Tube #: 1 2 3 4 5 6 7 8 9 10 11 12 13 14 15 16 17 18 19 20 21 22 23 24 25 26 27
100.0 % Buffer B
2000
669
474
50.0
0.0
1.00
0.75
0.50
0.25
0.00
500.0
400.0
300.0
200.0
100.0
0.0
-100.0
AU
0.00
5.00
10.00
15.00
20.00
25.00
Milliliters
mS/cm

B.

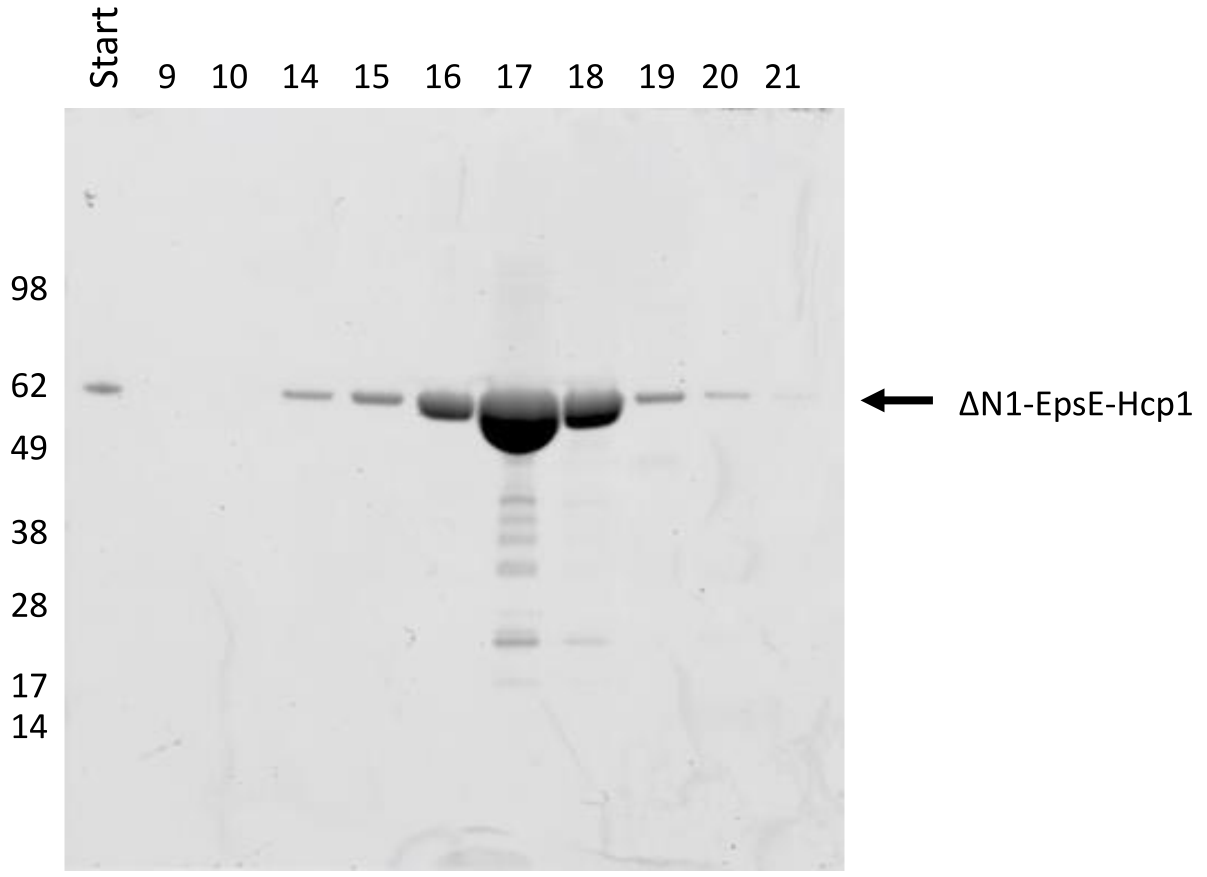

Supplementary Figure 4. Purification of hexameric ΔN1-EpsE-Hcp1. A. ΔN1-EpsE-Hcp1 was purified by metal affinity chromatography and then subjected to gel filtration using a Superose 6 column. Stars represent protein standards with sizes in kDa indicated above. **B**. Fractions containing protein peaks were analyzed via SDS-PAGE and proteins visualized with Coomassie staining. Fraction 17 was used for further analyses. The size of ΔN1-EpsE-Hcp1 is indicated with an arrow.
